# Cancer-associated fibroblasts: dual roles from senescence sentinels to death regulators and new dimensions in therapy

**DOI:** 10.3389/fimmu.2025.1635771

**Published:** 2025-07-18

**Authors:** Guixiang Ruan, Xiang Wang, Huiyi Ou, Duancheng Guo

**Affiliations:** ^1^ Department of Radiology, First People’s Hospital of Linping District, The Second Affiliated Hospital of Zhejiang University School of Medicine, Hangzhou, China; ^2^ Department of Pancreatic Surgery, Fudan University Shanghai Cancer Center, Shanghai, China; ^3^ Department of Oncology, Shanghai Medical College, Fudan University, Shanghai, China; ^4^ State Key Laboratory of Bioreactor Engineering, Shanghai Key Laboratory of New Drug Design, and School of Pharmacy, East China University of Science and Technology, Shanghai, China

**Keywords:** cancer-associated fibroblasts, cell senescence, cell death, SASP, TME

## Abstract

Cancer-associated fibroblasts (CAFs) are critical components of the tumor microenvironment (TME), playing a pivotal role in tumor initiation, progression, and therapeutic resistance. This review explores the dual roles of CAFs in regulating tumor cell senescence and cell death, elucidating their mechanisms in inducing cellular senescence, shaping an immunosuppressive milieu, and modulating cell death pathways. CAFs promote tumor progression by secreting pro-inflammatory factors and extracellular matrix (ECM) components, while also contributing to metabolic reprogramming, immune evasion, and therapy resistance, thereby influencing anti-cancer treatment efficacy. Studies indicate that the heterogeneity and plasticity of CAFs determine their distinct functions across various tumor types. Consequently, precision-targeted therapeutic strategies against CAFs, including the elimination of senescent CAFs, inhibition of the senescence-associated secretory phenotype (SASP), and disruption of CAF-mediated cell death evasion mechanisms, have emerged as promising directions in cancer research. This review provides a comprehensive analysis of CAFs functions and their potential as therapeutic targets, offering valuable insights into the development of novel anti-cancer strategies.

## Graphical Abstract

Relationship of CAFs in cell death and cellular senescence.CAFs play an important role in maintaining cell senescence and cell death and promote tumor progression, chemotherapy resistance, and immunosuppression.

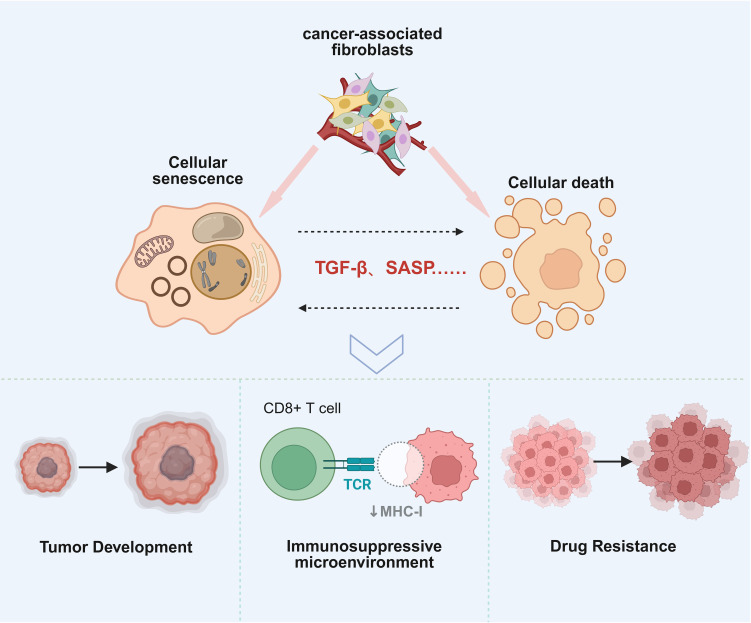

## 1 Introduction

TME plays a critical role in the initiation, progression, and metastasis of tumors, with CAFs being one of the most prominent stromal cell types in the TME (1). CAFs promote tumor proliferation, invasion, metastasis, and immune evasion by secreting various cytokines, exosomes, and ECM components, which interact with tumor cells and other TME components (2). Recent studies have also demonstrated that CAFs play a key role in tumor metabolic reprogramming, angiogenesis, and chemotherapy resistance (3, 4).CAFs regulate the biological behavior of tumor cells directly or indirectly by secreting signaling molecules such as transforming growth factor-beta (TGF-β), interleukin-6 (IL-6), and vascular endothelial growth factor (VEGF) (5, 6). For example, TGF-β not only induces epithelial-mesenchymal transition (EMT) but also promotes tumor cell invasiveness through the activation of the Smad signaling pathway (7). Moreover, CAFs remodel the ECM, forming physical barriers that limit immune cell infiltration, thereby aiding tumor cells in evading immune surveillance (8). Recent research has found that the heterogeneity of CAFs is a crucial factor underlying their complex roles in tumors. Based on their phenotypes and functions, CAFs can be classified into tumor-promoting and tumor-suppressing subpopulations. Tumor-promoting CAFs promote tumor progression by secreting pro-inflammatory factors and ECM components, whereas tumor-suppressing CAFs may inhibit tumor growth by inducing immune responses (9). Additionally, CAFs are closely associated with the efficacy of immune checkpoint inhibitors, and therapeutic strategies targeting CAFs or their secreted factors have emerged as a new direction in cancer treatment (10).

Cellular senescence is a stable state of cell cycle arrest that plays a dual role in tumor initiation and progression (11). On one hand, cellular senescence is considered a tumor-suppressive mechanism, preventing tumor formation by halting the proliferation of damaged or premalignant cells (12). On the other hand, senescent cells secrete a SASP, which releases a variety of pro-inflammatory factors and matrix remodeling proteins, exacerbating the TME and thereby accelerating tumor progression (13). For example, cytokines in the SASP, such as interleukin-6 (IL-6), interleukin-8 (IL-8), and matrix metalloproteinases (MMPs), can induce angiogenesis, EMT, and immune suppression, creating favorable conditions for tumor cell invasion and metastasis (14).Recent studies have shown a close relationship between CAFs and cellular senescence. In the TME, CAFs are not only one of the main sources of SASP but also important responders to SASP signaling (15). Senescent CAFs exacerbate TME fibrosis and inflammation by secreting high levels of ECM components and pro-inflammatory factors, promoting tumor malignant transformation (16). Furthermore, research has found that senescent CAFs can induce tumor cell senescence through paracrine signaling, forming a “senescence-promoting cancer” positive feedback loop (17). For instance, TGF-β and IL-1β secreted by senescent CAFs can induce tumor cell senescence while enhancing their invasiveness and chemotherapy resistance (18).Notably, therapeutic strategies targeting CAFs and cellular senescence are emerging as new directions in cancer treatment. By clearing senescent CAFs or inhibiting SASP secretion, the immune-suppressive state of the TME can be significantly improved, enhancing the efficacy of chemotherapy and immunotherapy (19, 20).

Cell death is a crucial biological process for maintaining tissue homeostasis and eliminating abnormal cells, and it plays a dual role in tumor initiation and progression. On one hand, cell death, especially programmed cell death (such as pyroptosis, ferroptosis, and necroptosis), can effectively eliminate premalignant cells, thus preventing tumor formation (21, 22). On the other hand, tumor cells can gain survival advantages by escaping cell death mechanisms, which facilitates tumor progression and the development of resistance (23).In the TME, there is a complex interaction between CAFs and cell death processes (24). CAFs significantly influence the fate of tumor cell death by secreting cytokines, exosomes, and metabolic products (25, 26). Additionally, CAFs regulate the redox state in the TME, affecting the sensitivity of tumor cells to ferroptosis (27). On the other hand, CAFs themselves may also undergo cell death, which further affects the dynamic balance of the TME (28). Studies have shown that the apoptosis or necroptosis of CAFs can lead to ECM remodeling and the release of inflammatory factors, thereby promoting tumor invasion and metastasis (29). Furthermore, CAFs’ pyroptosis, by releasing a large amount of pro-inflammatory cytokines, may trigger local inflammatory responses and create favorable conditions for tumor cell growth (30). Targeting the interaction between CAFs and cell death processes is emerging as a new direction in cancer therapy.

Given their role in both senescence regulation and signaling, CAFs function as key signaling intermediaries in the tumor microenvironment. By modulating these two critical processes, CAFs orchestrate the balance between tumor cell proliferation and death, influencing tumor aggressiveness and response to therapy. Their dual role as both promoters of senescence and inhibitors of cell death provides a complex challenge for therapeutic strategies aimed at targeting CAFs. Understanding these mechanisms offers new opportunities for overcoming tumor resistance and improving therapeutic outcomes. This review will summarize the role of CAFs in the tumor microenvironment, explore the relationship between CAFs and cellular senescence as well as cell death, and discuss the role of CAFs in the dynamic balance between senescence and cell death. It aims to actively uncover the complexity of CAFs in cancer and their potential as therapeutic targets.

## 2 The role and classification of CAFs in tumorigenesis and development

### 2.1 Classification of CAFs

As research on CAFs advances, the understanding of their various subtypes and their roles in tumor progression has deepened. Single-cell RNA sequencing has revealed distinct CAF subtypes, including myofibroblastic CAFs(myCAFs), inflammatory CAFs (iCAFs), and antigen-presenting CAFs (apCAFs), each exhibiting unique functional characteristics in different types of cancer.

#### 2.1.1 myCAFs

MyCAFs are a subset of CAFs characterized by a pronounced myofibroblastic phenotype. They primarily contribute to ECM remodeling and provide mechanical support within the tumor microenvironment. MyCAFs are distinguished by high expression of α-smooth muscle actin (α-SMA), which plays a key role in increasing tumor tissue stiffness and influencing both tumor growth and drug penetration (31). Studies have demonstrated that the enrichment of myCAFs in pancreatic ductal adenocarcinoma (PDAC) is strongly associated with increased tumor invasiveness and poor prognosis. Moreover, their presence contributes to the establishment of an immunosuppressive milieu, thereby inhibiting immune cell infiltration and tumor clearance (32).

#### 2.1.2 iCAFs

ICAFs are a highly secretory subtype that exhibit low α-SMA expression while producing elevated levels of pro-inflammatory cytokines, such as IL-6 and IL-1β. These cells promote tumor cell proliferation, resistance to apoptosis, and immune evasion (33). Notably, in head and neck squamous cell carcinoma (HNSCC), the CCR7/DUSP1 signaling axis regulates iCAFs activity, enhancing tumor cell growth potential (34). Furthermore, iCAFs activate the JAK/STAT3 signaling pathway, which facilitates the recruitment of myeloid-derived suppressor cells (MDSCs), further reinforcing the immunosuppressive tumor microenvironment.

#### 2.1.3 apCAFs

ApCAFs are a unique CAF subtype capable of expressing major histocompatibility complex class II (MHC-II) molecules, allowing them to interact with T cells and modulate antitumor immune responses (35) In pancreatic cancer, apCAFs have been widely studied, with findings indicating that their high expression of antigen-presenting molecules, such as HLA-DR, may interfere with conventional antigen presentation pathways, thereby weakening T cell-mediated antitumor immunity (34). Moreover, apCAFs have been shown to secrete TGF-β, promoting the expansion of regulatory T cells (Tregs) and further suppressing antitumor immune responses.

#### 2.1.4 Other CAF subtypes

Recent studies have identified additional CAFs subtypes with distinct roles in tumor progression. These include vascular-promoting CAFs (vCAFs), immunosuppressive CAFs (imCAFs), metabolism-regulating CAFs (mCAFs), and stem-like CAFs (scCAFs). In gastric cancer, vCAFs have been linked to abnormal tumor angiogenesis and chemotherapy resistance, primarily through the hypoxia-inducible factor-1 alpha (HIF-1α) pathway, which promotes tumor adaptation to hypoxic conditions and enhances resistance to anti-angiogenic therapies (36, 37).In breast and colorectal cancers, imCAFs are associated with increased tumor-infiltrating Tregs, highlighting their role in establishing an immunosuppressive tumor microenvironment (38). Similarly, scCAFs have been identified in bladder cancer and HNSCC, with evidence suggesting that they originate from partially dedifferentiated fibroblasts and promote tumor stemness via the Wnt/β-catenin signaling pathway (39).

**Figure 1:**
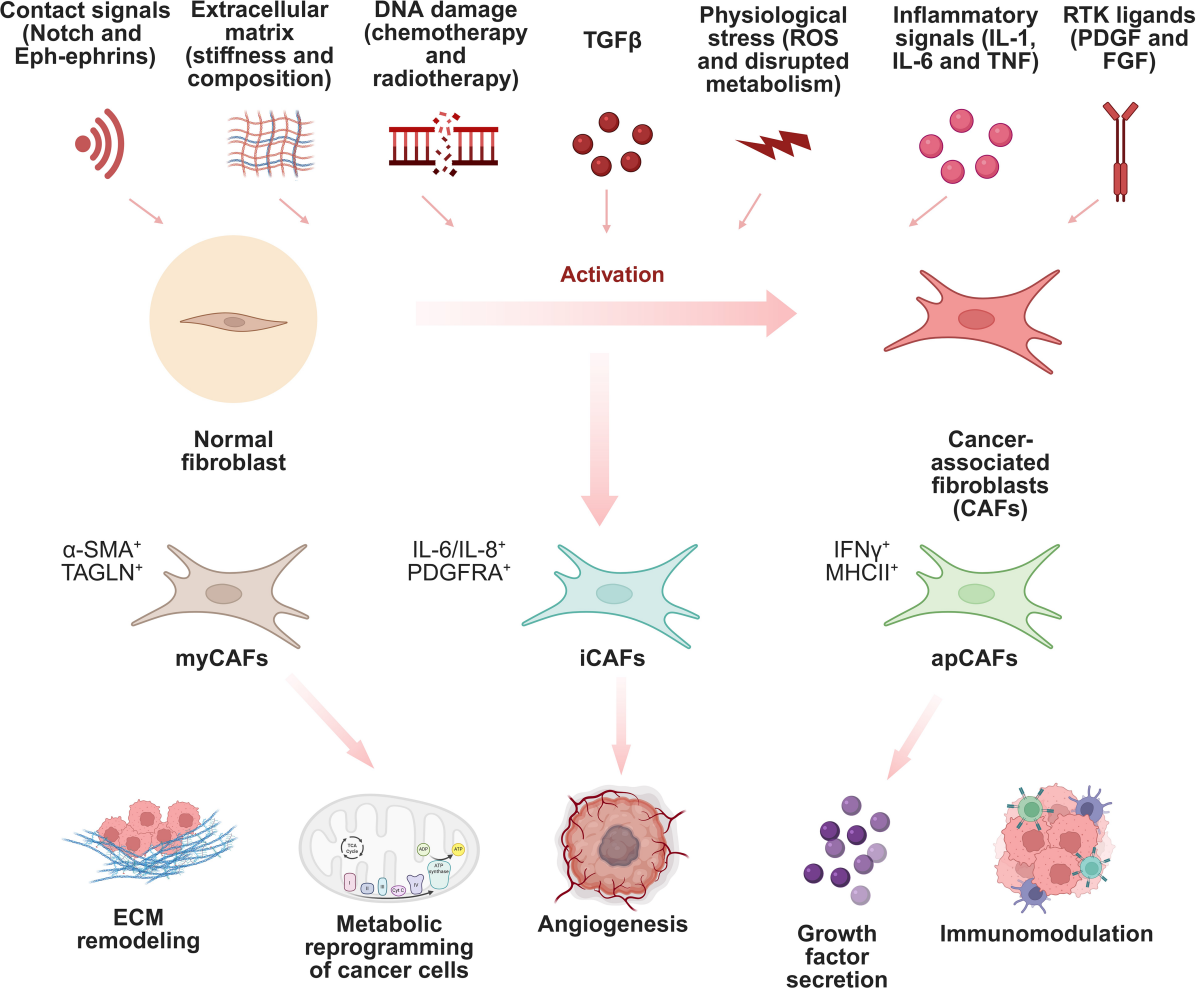
Classification and functions of different CAFs. This schematic highlights the multiple mechanisms that can lead to the activation of CAFs and the various types and functions of CAFs. Due to the wide variety of cell precursors and different activation mechanisms, CAFs show a high degree of heterogeneity and are generally divided into myCAFs, iCAFs, and apCAFs.

The spatial distribution of CAFs has been a key research focus. In hepatocellular carcinoma (HCC), single-cell and spatial transcriptomics have revealed distinct CAF differentiation trajectories, highlighting the prognostic significance of various fibroblast subtypes. Additionally, studies on cancer cell-CAFs interactions have led to the identification of novel molecular signatures that facilitate immune evasion and tumor progression (40).Overall, these findings underscore the complexity and heterogeneity of CAFs, reinforcing their potential as key regulators of the tumor microenvironment and promising therapeutic targets for improving cancer treatment outcomes (**Figure 1**).

### 2.2 Formation and functions of CAFs in the tumor microenvironment

CAFs originate from various sources, including normal fibroblasts, bone marrow-derived stromal cells, endothelial cells, and adipose-derived mesenchymal stem cells. Tumor-derived factors such as TGF-β, PDGF, and IL-6 trigger the activation of CAFs, endowing them with pro-tumorigenic functions. CAFs are highly heterogeneous and influence tumor progression through multiple interconnected mechanisms—including secretion of cytokines, remodeling of ECM, immune modulation, and metabolic reprogramming. For example, matrix metalloproteinases secreted by CAFs degrade the ECM to facilitate tumor invasion, while factors like CXCL12 promote angiogenesis. Additionally, CAFs impair anti-tumor immunity by inhibiting T-cell activation and recruiting regulatory T cells, fostering an immunosuppressive microenvironment.

The role and characteristics of CAFs vary by tumor type. In PDAC, CAFs dominate the stroma and contribute to chemoresistance primarily via the IL-6/JAK/STAT3 signaling axis. Gene profiling studies by Hu et al. identified nine distinct CAFs subtypes in PDAC, underscoring their functional diversity (40). In breast cancer, CAFs promote tumor progression by enhancing cell motility and activating the PI3K/AKT/mTOR metabolic pathway (41). In gastric cancer, the TGF-β signaling axis in CAFs is a key driver of EMT and immune evasion (42). Moreover, CAFs can engage in metabolic coupling by supplying metabolites such as lactate to fuel cancer cell growth (43). Beyond supporting tumor progression, CAFs also play a central role in resistance to therapy. They contribute to radiotherapy and chemotherapy resistance via TGF-β signaling and can transfer drug-resistance mediators (e.g., P-glycoprotein) through exosomes to neighboring cancer cells (44). These multifaceted functions highlight CAFs as critical components of the tumor microenvironment and promising therapeutic targets (45).

Recent research underscores the complex interplay between CAFs and immune cells, particularly dendritic cells (DCs), within the TME. DCs are pivotal for initiating anti-tumor immunity, yet CAFs actively drive immune evasion through multiple mechanisms. These include secreting TGF-β and IL-6, which impair DC maturation, antigen presentation, and cytokine production, ultimately compromising T cell priming (46, 47).Specific CAFs subpopulations, such as CAFs-S1 fibroblasts, further shape an immunosuppressive milieu by expressing ligands and chemokines that selectively recruit regulatory T cells (Tregs) while excluding immunostimulatory CD103^+^ DCs (48). More recent findings reveal that CAFs-derived exosomes and cytokines suppress DC differentiation and promote a tolerogenic state, thereby hindering antigen-specific T cell activation (49).Critically, targeting CAFs-secreted WNT2 has been shown to restore DC-mediated anti-tumor immunity, highlighting a promising therapeutic strategy for reactivating immune responses in CAF-rich tumors (25). Overall, the CAFs-DC axis, while still underexplored, represents a key target with significant translational potential for sensitizing tumors to immunotherapy.

### 2.3 CAF’s role in various leukemias

CAFs traditionally studied in the context of solid tumors, are increasingly recognized as pivotal players in the TME of hematologic malignancies. In diseases such as acute and chronic leukemias, multiple myeloma, and myeloproliferative neoplasms, CAFs influence tumor biology by supporting cancer cell survival, mediating immune evasion, and fostering drug resistance (50).

CAFs originate from diverse precursors, including mesenchymal stem cells (MSCs) and resident fibroblasts. Upon activation—often signaled by leukemic cells—they adopt enhanced proliferative and secretory capacities. They produce cytokines like IL-6 and GDF15 and contribute to remodeling the extracellular matrix, shaping a protective niche that impedes therapeutic efficacy. For instance, in acute myeloid leukemia (AML), CAFs have been shown to reduce chemosensitivity via secretion of GDF15 (51). Similarly, in B-cell acute lymphoblastic leukemia (B-ALL), CAF-like cells derived from MSCs amplify leukemia invasiveness through the SDF-1/CXCR4 axis (52).

Moreover, chronic lymphocytic leukemia (CLL) cells release exosomes that transform stromal cells into CAFs, perpetuating disease progression and therapeutic resistance (53). These findings underscore CAFs not only as contributors to leukemia pathogenesis but also as potential therapeutic targets. Inhibiting CAFs-related signaling pathways like TGF-β and JAK/STAT is already under preclinical and clinical exploration, offering hope for more effective, resistance-proof leukemia therapies (54).

## 3 CAFs and cell senescence

### 3.1 Related molecules and pathways that drive the senescence of CAFs

#### 3.1.1 DNA damage and epigenetic dysregulation

Chronic inflammation and chemotherapy/radiotherapy-induced DNA damage activate the ATM/ATR-Chk1/2 signaling axis, driving CAFs into senescence, as evidenced by the marked upregulation of cell cycle inhibitors p16INK4a and p21CIP1 (20, 55). This DNA damage response (DDR) pathway not only directly mediates cellular senescence but also modulates the tumor microenvironment through the SASP, which facilitates intercellular communication and matrix remodeling. Concurrently, epigenetic dysregulation in CAFs, particularly the loss of histone H3K27 trimethylation (H3K27me3), has been identified as a critical driver of pathological ECM stiffening in pancreatic cancer. Higashiguchi et al. demonstrated that H3K27me3 deficiency in CAFs derepresses pro-fibrotic genes (e.g., LOX and COL1A1), leading to excessive ECM deposition and biomechanical alterations (56). These findings underscore a synergistic interplay between DDR and epigenetic mechanisms in tumor microenvironment reprogramming: DDR activation initiates CAFs senescence, while epigenetic imbalance enhances chromatin accessibility at fibrogenic loci, collectively amplifying ECM remodeling and tumor progression.

#### 3.1.2 Metabolic reprogramming and hypoxic stress

Metabolic reprogramming and hypoxic stress play pivotal roles in the pro-tumorigenic functions of senescent CAFs. As demonstrated by Takasugi et al., mitochondrial dysfunction in senescent CAFs leads to aberrant accumulation of reactive oxygen species (ROS), which activates the NF-κB signaling pathway. This activation drives the secretion of pro-inflammatory cytokines such as IL-6 and IL-8, exacerbating chronic inflammation in the tumor microenvironment, thereby facilitating tumor progression and immune evasion (57). Conversely, hypoxic stress induces metabolic reprogramming in CAFs through stabilization of hypoxia-inducible factor 1α (HIF-1α). Studies reveal that HIF-1α upregulates insulin-like growth factor 1 (IGF1) secretion, which sustains the self-renewal capacity and stemness features of esophageal squamous cell carcinoma (ESCC) stem cells, ultimately promoting therapeutic resistance and tumor recurrence (58). These processes collectively illustrate the intricate interplay among metabolic reprogramming, hypoxia, and cellular senescence in driving cancer aggressiveness, highlighting their synergistic contribution to malignant transformation and stromal evolution.

### 3.2 Functional heterogeneity of senescent CAFs

#### 3.2.1 myCAFs

CAFs play a pivotal role in cancer progression through their diverse subpopulations, each with distinct functional characteristics. In breast cancer, A high-dimensional flow cytometry single-cell analysis revealed a unique subset of senescence-like TSPAN8+ myCAFs that is associated with chemotherapy resistance and poor survival outcomes in several breast cancer patient cohorts. These TSPAN8+ myCAFs enhance the stem-like properties of adjacent BC cells by secreting SASP-related factors, including IL-6 and IL-8, which help counteract the effects of chemotherapy. This phenomenon has been observed in patients, where these CAFs protect tumors by forming an extracellular matrix that shields them from treatment effects (59).

Studies have also identified specific subpopulations of myofibroblast CAFs that undergo senescence in mouse and human mammary tumors. The authors’ study showed that senescent CAFs secrete an extracellular matrix that inhibits the cytotoxic activity of natural killer (NK) cells, a key player in the immune system’s defense against tumors. By inhibiting NK cell function, senescent CAFs promote tumor growth. Elimination of senescent CAFs, either genetically or pharmacologically, restores NK cell-mediated cytotoxicity and significantly reduces tumor progression. In addition, studies have revealed that senescent CAFs are present in multiple breast cancer subtypes, including HER2+, ER+ and triple-negative breast cancers, as well as ductal carcinoma *in situ* (DCIS) (16). In these cases, the presence of senescent CAFs was associated with tumor recurrence, further confirming their role in cancer progression.

#### 3.2.2 iCAFs

In the tumor microenvironment, senescent CAFs exhibit a distinct secretory phenotype under stress conditions. This SASP contributes to cancer progression and chemoresistance. Studies have shown that in mouse models, inflammation-driven downregulation of EZH2 maintains SASP in CAFs by demethylating the H3K27me3 mark and enhances the formation of peritoneal tumors in gastric cancer (GC) through the JAK/STAT3 signaling pathway. JAK/STAT3 inhibitors prevent the increased viability of gastric cancer cells and peritoneal tumor formation induced by senescent CAFs. Single-cell mass spectrometry has revealed the presence of fibroblasts in the ascitic fluid of GC patients with peritoneal dissemination, showing high levels of p16 expression and SASP factors (17). Another study identified a group of iCAFs in rectal cancer patients, which are associated with poor responses to chemotherapy and radiotherapy. Using a mouse rectal cancer model and patient-derived tumor organoids and primary stromal cells, researchers found that interleukin-1α (IL-1α) not only induces the polarization of CAFs into an inflammatory phenotype after radiation but also triggers oxidative DNA damage, making iCAFs prone to therapy-induced senescence mediated by p53. This leads to resistance to chemoradiotherapy and disease progression. Inhibiting IL-1, preventing iCAFs senescence, or applying senolytic therapy makes the mice more sensitive to radiation (60). It has also been shown that p16highsenescent (p16h-sn) fibroblasts accumulate with age, constitute iCAFs and promote tumor growth in bladder cancer models (61). These findings contribute to a deeper understanding of the role of iCAFs-associated senescence in tumor progression and therapy.

### 3.3 Dynamic interactions between senescent CAFs and TME

#### 3.3.1 Senescent CAFs in tumor immune microenvironment

Senescent CAFs not only contribute to tumor progression but also mediate immunosuppression by altering the immune microenvironment through the analysis of cytokine secretion, affecting metastatic potential and therapeutic response. In diffuse-type gastric cancer (DGC), senescent CAFs promote peritoneal metastasis via IL-8-mediated crosstalk. The secreted IL-8 enhances DGC cell migration, thereby supporting peritoneal metastasis. *In vivo* models show that co-inoculation of senescent CAFs with DGC cells significantly promotes metastasis, a process that can be attenuated by blocking IL-8 receptors. This finding emphasizes the critical role of IL-8 in mediating the interaction between senescent CAFs and tumor cells, providing a mechanism for the increased metastatic potential of DGC (62).Similarly, studies have also shown that IL-8 plays a role in senescent CAFs, with researchers identifying that senescent CAFs in pancreatic cancer promote tumor cell invasion through IL-8 secretion, exhibiting distinct pro-cancer activity (63). Additionally, based on large-scale scRNA-seq datasets, researchers have established a fibroblast senescence-related signature, discovering that CDC6, by regulating TGF-β1 secretion and oxidative stress, promotes fibroblast senescence and affects immune checkpoint inhibitor (ICI) responses (64).

Senescent CAFs can also influence the tumor microenvironment by mediating immune cell behavior. In pancreatic cancer models, the elimination of senescent CAFs increases the proportion of activated CD8+ T cells, thereby enhancing the efficacy of immunotherapy. This finding indicates that senescent CAFs directly suppress immune cell function by secreting specific factors, thereby influencing tumor growth and responses to immunotherapy (65).Additionally, it has been shown that senescent CAFs can orchestrate immune evasion by secreting pro-inflammatory cytokines and factors associated with the SASP, including IL-6, IL-8, and TGF-β. These senescent fibroblasts can recruit and expand immunosuppressive cells such as Tregsand myeloid-derived suppressor cells (MDSCs), which in turn suppress the function of effector immune cells like cytotoxic T lymphocytes (CTLs) and NK cells (66).In breast cancer, senescent CAFs have also been shown to specifically inhibit the cytotoxic function of NK cells, promoting tumor growth. Clearing senescent CAFs enhances the killing capacity of NK cells, suppressing tumor growth (16).These findings emphasize the translational potential of targeting fibroblast senescence as a novel therapeutic strategy to mitigate immune resistance and enhance anti-tumor efficacy.

#### 3.3.2 Crosstalk between senescent CAFs and the extracellular matrix

Senescent CAFs significantly influence tumor progression through remodeling of the ECM, a complex and dynamic scaffold that regulates cell adhesion, migration, and signaling (67). In tumors such as pancreatic and esophageal cancers, senescent CAFs enhance ECM deposition and stiffness by secreting proteins like collagen and fibronectin, which contribute to a fibrotic and immunosuppressive microenvironment (68). This altered ECM not only facilitates cancer cell invasion and metastasis but also restricts immune cell infiltration, particularly of cytotoxic cells like NK cells and CD8^+^ T cells, thereby undermining anti-tumor immunity (15).

Additionally, senescent CAFs contribute to chemoresistance and immune evasion by releasing cytokines and matrix metalloproteinases (MMPs), which further modify the ECM composition (69, 70). These changes create both a physical and biochemical barrier that impedes drug delivery and immune-mediated tumor clearance (71). As such, therapeutic strategies aimed at targeting senescent CAFs-derived ECM components or modulating their activity hold promise in improving the efficacy of cancer therapies by restoring immune access and enhancing treatment penetration.

### 3.4 Precision therapeutic strategies targeting senescent CAFs

The role of senescent CAFs in cancer progression has garnered increasing attention, particularly in the context of targeted therapies. Research has demonstrated the development of engineered cancer cell-mimetic nanoparticles designed for dual-targeting the elimination of CAFs and senescent CAFs (73). These nanoparticles, loaded with ABT-263 targeting FAP+ senescent CAFs, can directly suppress the pro-tumorigenic effects of senescent CAFs. Additionally, they prolong blood circulation, enhance the radiation resistance of acquired and patient-derived radio-resistant tumor cells, and effectively exert anti-tumor effects. This strategy has the potential to reverse the tumor immunosuppressive microenvironment and boost systemic anti-tumor immunity, thereby enhancing the radiosensitivity of breast cancer to radiotherapy.

In lung cancer, the application of FOXO4-DRI, which targets the apoptosis of senescent-like fibroblasts, enhances the sensitivity to radiotherapy while alleviating radiation-induced pulmonary fibrosis *in vivo (* 20). Furthermore, a study has shown that a combination of senolytic drugs, dasatinib and quercetin, can clear p16+ senescent CAFs in pancreatic cancer, thereby impacting tumor progression (74).

Another study identified that senescent CAFs secrete a decoy protein that interferes with the cytotoxic activity of activated T effector cells (Teff). In a matrix-rich pancreatic cancer mouse model, the administration of an antibody blocking the decoy protein led to the recalibration of stromal fibroblasts, reducing the proportion of senescent CAFs (75). This resulted in enhanced tumor-infiltrating T cells and improved effector function, effectively counteracting tumor progression.

**Figure 2:**
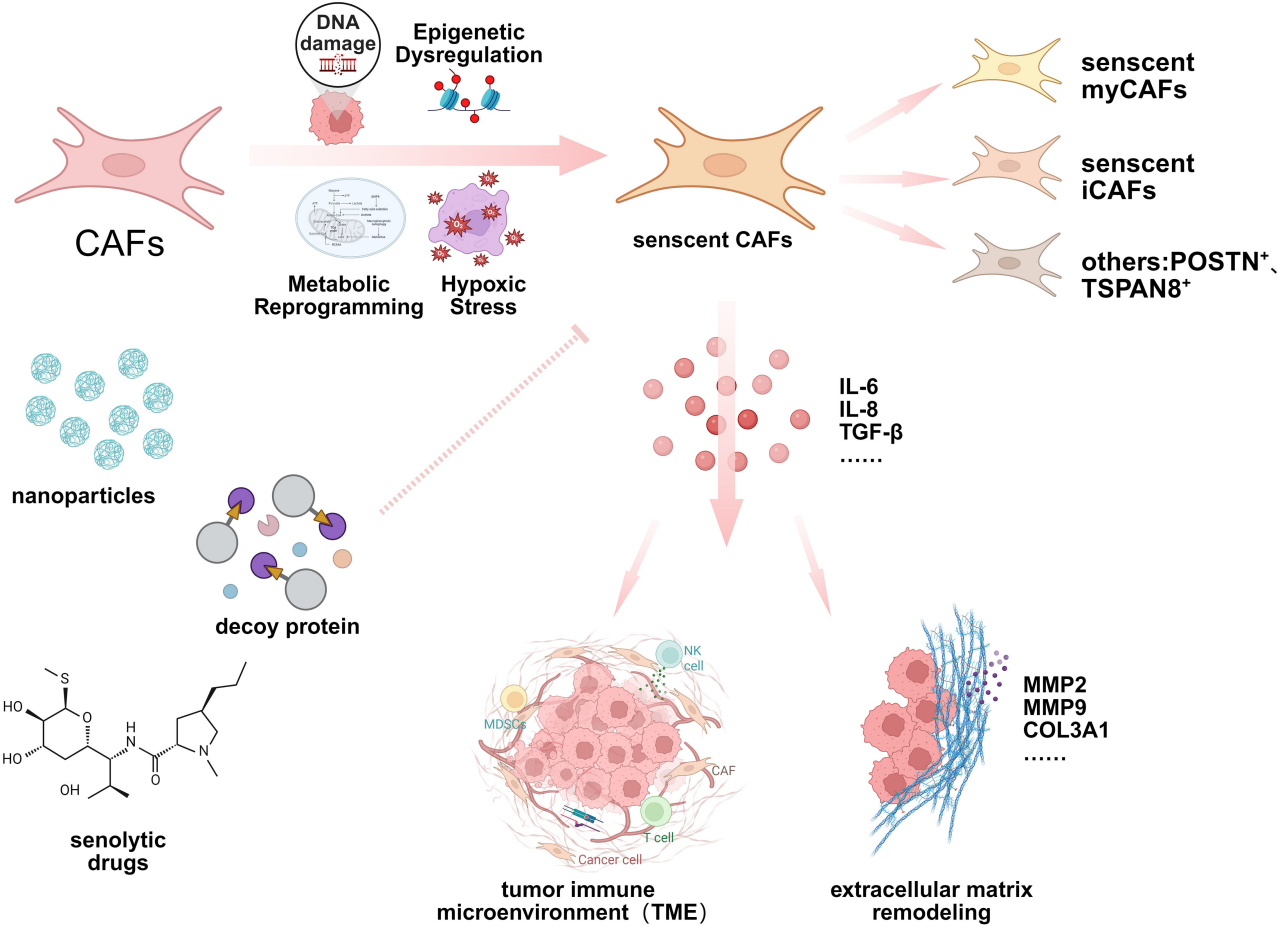
Activation mechanism, function and targeting strategy of senescent CAFs. This schematic diagram highlights the formation mechanism and heterogeneity of senescent CAFs. Senescent CAFs affect processes such as TME and ECM remodeling by secreting multiple cytokines. Senescent CAFs can be targeted for treatment through various means such as senolytic drugs, nanoparticles, and decoy proteins.

Targeting senescent CAFs is a promising therapeutic strategy that can enhance tumor response to immunotherapy and conventional treatments. By addressing the immunosuppressive and pro-metastatic roles of these fibroblasts, precision therapies may offer new avenues to overcome treatment resistance and improve patient prognosis (**Figure 2**).

## 4 The role of CAFs in different types of cell death forms

### 4.1 Apoptosis in CAFs

Apoptosis serves as a fundamental regulatory mechanism in tissue homeostasis, and aberrant apoptotic signaling contributes to tumor progression by preventing the clearance of malignant cells. Targeting apoptotic pathways to reshape the threshold of cancer cell death has emerged as a key therapeutic strategy in cancer (76–78). CAFs dynamically regulate the apoptotic sensitivity of adjacent tumor cells through the secretion of soluble mediators and interactions with the tumor microenvironment.

CAFs can modulate tumor cell apoptosis through direct cell–cell contact or the secretion of bioactive factors. In gastric cancer, CAFs have been shown to induce apoptosis via direct contact, a process mediated by the DR4-caspase-8 signaling pathway, which also governs tumor invasion patterns (79). Conversely, in OSCC, CAFs interact with apoptotic cancer cells to promote tumor proliferation via the STING signaling pathway (80). In CRC, CAF-derived exosomes transfer miRNAs and circRNAs to tumor cells, acting as inhibitors of apoptosis and thereby promoting CRC pathogenesis (81–84). These findings suggest that CAFs exhibit dual functional roles in apoptosis regulation, either promoting or inhibiting tumor cell death depending on the tumor context.

Chemotherapy primarily exerts its antitumor effects by inducing apoptosis in cancer cells; however, the presence of CAFs can diminish this apoptotic sensitivity. For instance, in ovarian cancer, CAFs secrete stromal cell-derived factor 1α (SDF-1α), facilitating tumor metastasis and enhancing apoptotic resistance (85). Additionally, CAFs activate the STAT3 signaling pathway to attenuate cisplatin-induced apoptosis, contributing to chemoresistance in ovarian cancer (86). Given these findings, targeting CAFs apoptosis has emerged as a promising therapeutic strategy. Studies have shown that curcumin can induce CAFs apoptosis via a ROS-mediated endoplasmic reticulum stress pathway (87). Moreover, cinnamaldehyde has been reported to induce endogenous apoptosis in prostate cancer-associated fibroblasts by disrupting glutathione-associated mitochondrial function, offering new perspectives on CAF-targeted therapy (88).

CAFs influence tumor cell apoptosis through multiple mechanisms while also exhibiting apoptotic resistance themselves, thereby impacting cancer progression and therapeutic response. Understanding the precise roles of CAFs in apoptosis regulation will be crucial for developing novel interventions that enhance treatment efficacy in cancer patients.

### 4.2 Ferroptosis in CAFs

CAFs play a critical role in regulating various forms of cell death, including ferroptosis, within the TME (89). Ferroptosis is a form of regulated cell death characterized by iron-dependent lipid peroxidation, and recent research has begun to explore how CAFs influence ferroptosis, affecting tumor progression and therapy responses (90, 91).

#### 4.2.1 Exosomal delivery of non-coding RNAs by CAFs suppresses ferroptosis

CAFs deliver a variety of functional RNA molecules via exosomes, forming an intercellular network that inhibits ferroptosis. In gastric cancer, exosomal miR-522 secreted by CAFs targets ALOX15, blocking lipid peroxidation and ROS accumulation, thereby significantly suppressing ferroptosis in cancer cells (24). In prostate cancer, exosomal miR-432-5p silences CHAC1, a key oxidative stress gene, weakening ferroptosis and driving chemotherapy resistance (92). In breast cancer and glioblastoma, CAFs disrupt the ferroptosis pathway by secreting miR-454-3p (targeting lipid metabolism enzyme ACSL4) and upregulating lncRNA DLEU1, promoting tumor survival (93, 94). Additionally, in colorectal cancer, CAFs regulate the m6A modification of ACSL3 via exosomal METTL3, and in lung cancer, the ROR1-AS1/IGF2BP1 axis stabilizes the expression of SLC7A11, both significantly inhibiting ferroptosis and enhancing metastatic potential (95, 96). These studies highlight exosomes as a core mediator through which CAFs regulate ferroptosis, and the RNA molecules they carry may serve as novel targets for reversing drug resistance.

#### 4.2.2 CAF-mediated metabolic reprogramming and signal pathways cooperatively drive ferroptosis resistance

CAFs reshape the metabolic microenvironment and activate key signaling pathways to build a ferroptosis-resistant barrier in tumors. In pancreatic cancer, CAFs reprogram cysteine metabolism to elevate antioxidant levels, such as glutathione, within tumor cells, thereby enhancing resistance to ferroptosis (97). In oral squamous cell carcinoma (OSCC), PDPN+ CAFs activate the FTX/FEN1/ACSL4 signaling axis, suppressing lipid peroxidation and promoting an invasive phenotype (98). These mechanisms suggest that targeting CAF-mediated metabolic enzymes (e.g., cysteine transporter xCT) or signaling nodes could disrupt the tumor-stroma interaction, restore ferroptosis sensitivity, and improve chemotherapy efficacy.

#### 4.2.3 CAF-mediated ferroptosis regulation and chemotherapy resistance

CAFs directly contribute to chemotherapy resistance by inhibiting tumor ferroptosis. In pancreatic cancer, exosomal ACSL4 secreted by CAFs promotes polyunsaturated fatty acid metabolism, significantly reducing the sensitivity of tumor cells to chemotherapy drugs such as gemcitabine (27). In gastric cancer, CAFs-derived DACT3-AS1, a long non-coding RNA, establishes an oxaliplatin-resistant phenotype through epigenetic regulation (99). In nasopharyngeal carcinoma treatment, CAFs-secreted FGF5 induces a ferroptosis-suppressive microenvironment via the FGFR2/Nrf2 signaling cascade, limiting the therapeutic effect of cisplatin. Targeting the FGF5/FGFR2 axis could offer a strategy to reverse cisplatin resistance in this context (100).

#### 4.2.4 Dual role of CAFs in ferroptosis regulation

Recent research reveals that CAFs have a dual role in regulating ferroptosis. In the gastric cancer microenvironment, CAFs upregulate iron transport proteins ferroportin1 and hephaestin, leading to the abnormal accumulation of unstable iron pools in NK cells. This results in ferroptosis-dependent immune dysfunction, which weakens NK cell-mediated tumor cytotoxicity. Experimental evidence shows that dual intervention with iron chelators and FSTL1-neutralizing antibodies can effectively restore NK cell toxicity, offering a new strategy for improving tumor immunotherapy outcomes (28). These findings underscore the complex role of CAFs in regulating ferroptosis and their influence on cancer progression and therapy resistance. By targeting CAFs and their secreted factors, new therapeutic approaches can be developed to enhance the effectiveness of cancer treatments, particularly in overcoming chemotherapy and immunotherapy resistance.

### 4.3 Pyroptosis in CAFs

CAFs play a crucial role in the TME and influence tumor progression through multiple mechanisms. Among these, the interaction between CAFs and pyroptosis has emerged as a research hotspot. Pyroptosis is an inflammatory form of programmed cell death mediated by the cleavage of gasdermin D (GSDMD) and is closely associated with inflammasome activation (101–103).

Recent studies have demonstrated that CAFs regulate pyroptosis-related gene expression, thereby influencing both their own pyroptotic process and that of tumor cells. For instance, single-cell trajectory analysis revealed that key pyroptosis-related genes such as CHMP6 and PLCG1 are expressed at various developmental stages of CAFs, suggesting that CAFs may dynamically modulate the tumor microenvironment through pyroptotic pathways (104). Additionally, CAFs may regulate pyroptosis to impact tumor progression. In breast cancer, inflammasome-activated CAFs have been found to influence tumor growth and invasion through pyroptotic pathways (105).In non-small cell lung cancer (NSCLC), the pyroptosis status of CAFs has been correlated with patient prognosis, indicating its potential as a therapeutic target (106). Within the breast cancer microenvironment, damage-associated molecular patterns (DAMPs) can activate the NLRP3 inflammasome, triggering pyroptosis in CAFs via the NLRP3/caspase-1/GSDMD signaling axis. This process leads to the release of interleukin-1β (IL-1β), which drives tumor progression and metastasis through paracrine signaling (107).

Notably, there is currently a lack of systematic studies on the molecular mechanisms by which CAFs directly regulate pyroptosis in tumor cells. Public data analyses suggest that the infiltration levels of CAFs in tumor tissues are positively correlated with GSDMD and GSDME expression in malignant cells, implying that CAFs may mediate pyroptosis in tumor cells through specific molecular crosstalk (108, 109). The role of pyroptosis in the TME exhibits dual characteristics. On one hand, it can suppress tumor progression by triggering immune responses. On the other hand, under certain conditions, CAFs may modulate pyroptotic pathways to prevent tumor cell death, thereby contributing to cancer resistance and progression (110).

From a therapeutic perspective, modulation of pyroptosis in CAFs presents a dual-edged opportunity. Targeted induction of pyroptosis in pro-tumorigenic CAFs may disrupt their stromal and immunosuppressive functions. For instance, Gao et al. found that caspase-1–dependent pyroptosis could convert αSMA^+^ CAFs into collagen-III^high^ iCAFs, which subsequently support chemoresistant cancer stem cells—highlighting potential risks of maladaptive CAFs reprogramming (111). On the other hand, Wang et al. reported that targeted pyroptosis induction in immunosuppressive CAFs led to enhanced T cell infiltration and tumor regression, especially when combined with immune checkpoint blockade (110).Emerging therapeutic strategies include the use of “CAFs-sensitizing agents” to selectively prime pyroptosis pathways, as well as nanocarrier systems for localized delivery of inflammasome activator (30). Despite this promise, challenges remain in defining CAF subtype-specific responses and avoiding pro-tumor inflammatory side effects.

**Figure 3:**
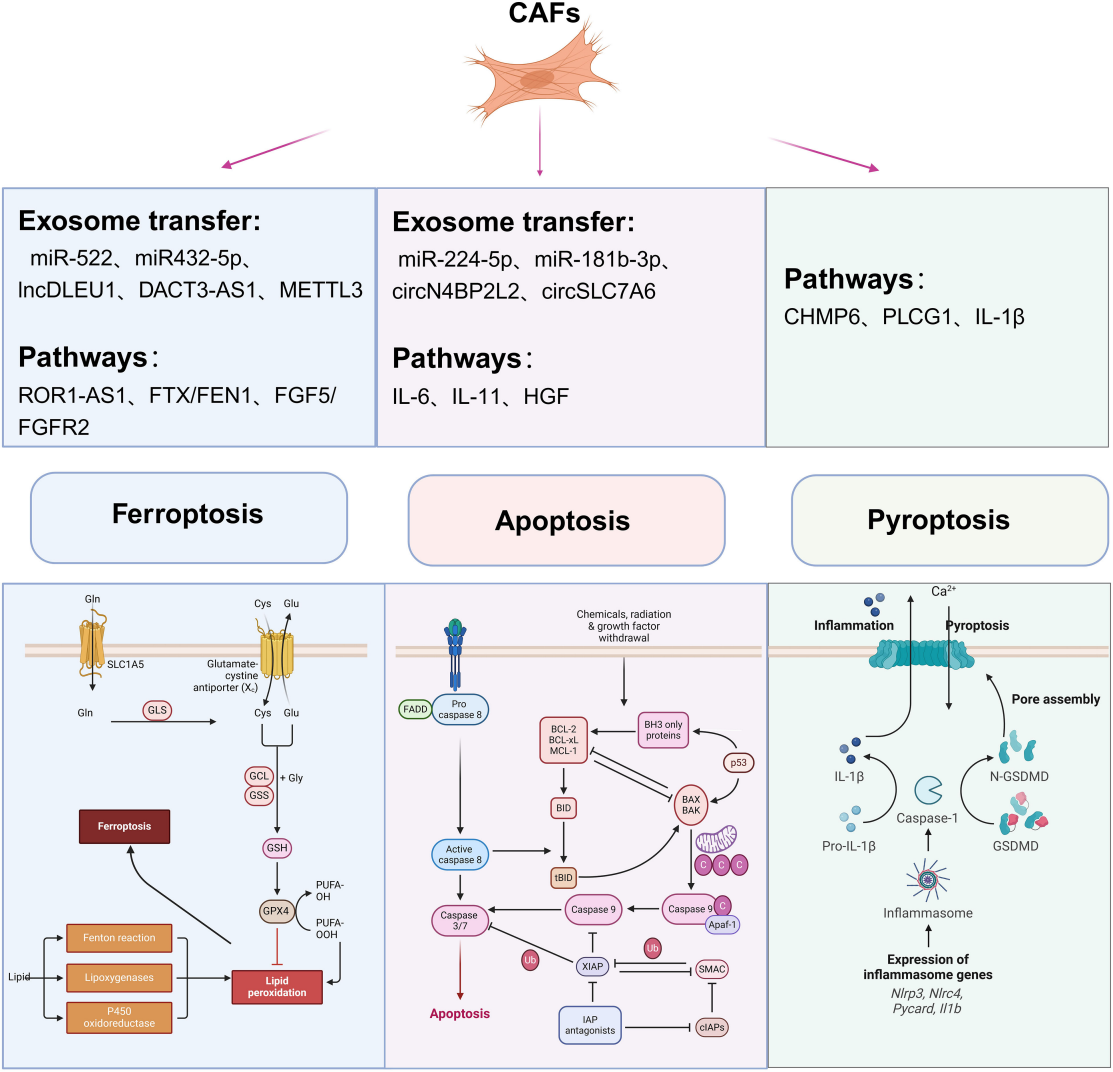
Role of CAFs in ferroptosis, apoptosis and pyroptosis. The core molecular mechanisms of different types of cell death and potential CAFs-derived molecules that regulate cell death. The various death forms, including apoptosis, ferroptosis, and pyroptosis, are described in detail, and the relationship between cell death and CAFs is explained.

Overall, CAFs play a complex role in pyroptosis regulation, as they can either promote pyroptosis in tumor cells or inhibit it to sustain cancer cell survival. However, the intricate mechanisms underlying this process remain to be fully elucidated. Further research into CAFs-mediated pyroptotic pathways could provide valuable insights for developing novel pyroptosis-based anticancer therapies (**Figure 3**).

## 5 CAFs as pivotal regulators of cellular senescence and death in TME

CAFs play a pivotal role in the TME, and accumulating evidence suggests that CAFs not only promote the accumulation of senescent cells but also regulate tumor cell apoptosis and proliferation through their secreted factors. As a result, CAFs may serve as a crucial bridge in the transition between cellular fates. This hypothesis is supported by the multifunctional nature of CAFs, including their involvement in intercellular communication, oxidative stress regulation, metabolic reprogramming, and the secretion of bioactive factors.

CAFs are considered part of the senescent cell population and exert their influence on tumor cells and the surrounding microenvironment through the SASP. Studies have demonstrated that the SASP of CAFs contributes to immunosuppression and affects the homeostasis of the TME (112). Additionally, CAFs regulate cell death and senescence through redox control and metabolic reprogramming. For instance, redox signaling between CAFs and cancer cells has been implicated in tumor resistance and increased oxidative stress in the extracellular environment (113). Moreover, CAFs can secrete lactate or other metabolites that alter the metabolic state of tumor cells, enabling their adaptation to hypoxic conditions and consequently delaying cell death (114, 115).

Mechanistically, TGF-β family plays a crucial role in balancing CAFs-mediated regulation of cell death and senescence. TGF-β signaling modulates CAFs activation and exhibits dual functionality in cellular senescence: on the one hand, it induces senescence, while on the other, it may sustain cell survival by inhibiting key apoptotic signals (116–118). Furthermore, CAFs influence apoptotic pathways by regulating molecules associated with programmed cell death, such as PD-L1, thereby contributing to immune evasion and the intricate interplay between senescence and apoptosis (72).

Under the regulatory influence of CAFs, senescence and apoptosis may represent a dynamic continuum rather than discrete events. The accumulation of senescent cells often exacerbates the inflammatory microenvironment, which, in turn, can trigger cell death. Thus, CAFs may serve as a critical intersection between these interconnected cellular processes.

## 6 Diagnosis and treatment of CAFs in tumor

### 6.1 Imaging diagnosis of CAFs

CAFs are widely distributed within TME and play a crucial role in tumor progression, invasion, and metastasis. Their unique phenotypic characteristics make them important targets for tumor imaging diagnostics. In recent years, researchers have developed various imaging diagnostic strategies by targeting CAF-specific biomarkers, such as fibroblast activation protein (FAP), microfibrillar-associated protein 5 (MFAP5), andα-SMA. These biomarkers are expressed across multiple tumor types, demonstrating their broad applicability. For example, Wang et al. investigated a novel gadolinium-based FAPI dimer molecular probe designed for tumor imaging via FAP targeting, which exhibited excellent safety and imaging contrast properties (119). Additionally, Fersing et al. reviewed the applications of FAP in nuclear medicine imaging, emphasizing its value in both diagnosis and treatment guidance (120). These studies collectively confirm the potential of CAF-targeted imaging diagnostics in oncology.

Recent years have witnessed significant progress in molecular imaging technologies for diagnosing CAFs. Various modalities, including positron emission tomography (PET), magnetic resonance imaging (MRI), computed tomography (CT), and optical imaging techniques (such as near-infrared fluorescence imaging), have been employed in CAF-related research. For instance, Fan et al. utilized ^68^Ga-OncoFAP microPET/CT imaging in a breast cancer model and demonstrated that this probe could selectively identify CAFs, thereby improving imaging precision (121). Furthermore, Gao et al. found that MFAP5+ CAFs contributed to the manifestation of extramural venous invasion (EMVI) in gastric cancer as detected by CT imaging, offering a novel imaging-based reference for early diagnosis (122). Moreover, Meng et al. proposed a multimodal imaging strategy that integrates PET and MRI to enhance the accuracy of CAFs detection (123). Collectively, these studies highlight the ongoing improvements in imaging techniques tailored for CAF-related tumor diagnostics.

Despite the substantial progress made in CAFs-targeted imaging diagnostics, several challenges remain in their clinical translation. One major obstacle is the heterogeneity of CAFs across different tumor types, leading to variability in biomarker expression and limiting the broad applicability of imaging agents. Additionally, some FAP-targeting imaging probes still suffer from issues such as metabolic instability and non-specific uptake. To address these concerns, Yu et al. suggested the optimization of FAP-based radiopharmaceuticals to enhance specificity and imaging contrast (124). Furthermore, the clinical feasibility and accuracy of PET/CT and MRI combinatorial imaging strategies require validation through large-scale clinical studies. Hilmi et al. noted that while FAP-PET holds promise for early pancreatic cancer diagnosis, extensive patient data are needed to substantiate its diagnostic efficacy (125). Looking ahead, the integration of artificial intelligence (AI) with imaging analysis is expected to refine CAFs imaging, leading to more accurate diagnostics and the development of personalized therapeutic strategies.

### 6.2 Therapeutic strategies targeting CAFs

The high plasticity and heterogeneity of CAFs present multiple opportunities for targeted therapy. Several approaches have been proposed, including the inhibition of FAP, immune-modulating therapies, and the use of CAFs-specific nanocarriers for drug delivery. Studies have shown that CAFs promote resistance to EGFR inhibitors via a CTHRC1-mediated metabolic feedback loop, and disrupting this pathway can enhance the efficacy of targeted therapies (126). Another promising strategy involves CD70-directed chimeric antigen receptor (CAR) NK cell therapy, which selectively targets CD70+ CAFs and has demonstrated therapeutic potential in colorectal and pancreatic cancer models (127). Additionally, FAP-targeted CAR-T cell immunotherapy has been reported to effectively eliminate CAFs within the tumor microenvironment, leading to significant anti-tumor effects (128). Another CAFs-targeting strategy focuses on inhibiting their tumor-promoting metabolic activity. CAFs contribute to tumor progression by supplying lactate through glycolytic metabolism, which fuels tumor cell metabolic reprogramming. Therefore, blocking CAF-driven glycolysis using LDH (lactate dehydrogenase) or MCT (monocarboxylate transporter) inhibitors could weaken their tumor-supportive role (129). Furthermore, CAFs play an immunosuppressive role in the tumor microenvironment, prompting researchers to explore immune checkpoint blockade (ICB) in combination with CAFs-targeting approaches, such as CAFs-CAR-T therapy, to enhance anti-cancer immune responses (130) (**Table 1**).

**Table 1:** Some drugs targeting CAF.

Drug/Strategy	Targeted Cancer Type	Description	Specific Target	Reference
FAP-targeted therapies	Pancreatic cancer, other solid tumors	FAP is highly expressed in CAFs and is a classical target	FAP	(132)
Tocilizumab	Pancreatic cancer, colorectal cancer	Targets IL-6 secreted by inflammatory CAFs	IL-6	(133)
Galunisertib	Pancreatic cancer	Suppresses CAF activation and balances CAF subtypes	TGF-β	(134)
AMD3100	Pancreatic cancer, breast cancer and prostate cancer	CAFs secrete CXCL12 promoting immune evasion	CXCL12/CXCR4	(135)
Vismodegib	Prostate and breast cancer	Inhibits Hedgehog signaling in CAFs	SMO, GLI	(136)
Simlukafusp alfa	Breast cancer, melanoma	Delivers immune factors targeted at CAFs	FAP	(137)
Navitoclax	Colorectal and lung cancer	Enhances drug sensitivity of tumor cells	Bcl-2/Bcl-xL	(138)
Gold Nanoparticles (AuNPs)	Pancreatic cancer, breast cancer	Functionalized AuNPs target CAFs for chemotherapy drug or RNA delivery, often combined with photothermal therapy to enhance efficacy	e.g., FAP	(139)
Iron Oxide Nanoparticles (FeNPs)	Lung cancer, breast cancer	Target the TGF-β pathway in CAFs to release anti-fibrotic agents, reduce CAF density	TGF-β	(139)

Recent studies indicate that CAFs play a key role in regulating tumor cell senescence and apoptosis. Standard cancer therapies, such as radiotherapy and chemotherapy, can induce senescent CAFs, which paradoxically promote tumor cell survival and drive disease progression (15). In response to this challenge, novel therapeutic strategies have been developed to target senescent CAFs and disrupt their pro-tumorigenic functions. One such approach is senolytic therapy, which selectively eliminates senescent CAFs, thereby improving cancer treatment efficacy (74). Another strategy involves inhibiting SASP, a pro-inflammatory secretome produced by senescent CAFs that enhances tumor growth and therapy resistance. By modulating senescent CAFs and their secretory activity, these approaches aim to attenuate their tumor-supportive roles and enhance the effectiveness of cancer treatments. Such interventions hold promise for personalized anti-cancer therapies, particularly in tumors with a strong CAFs influence.

**Figure 4:**
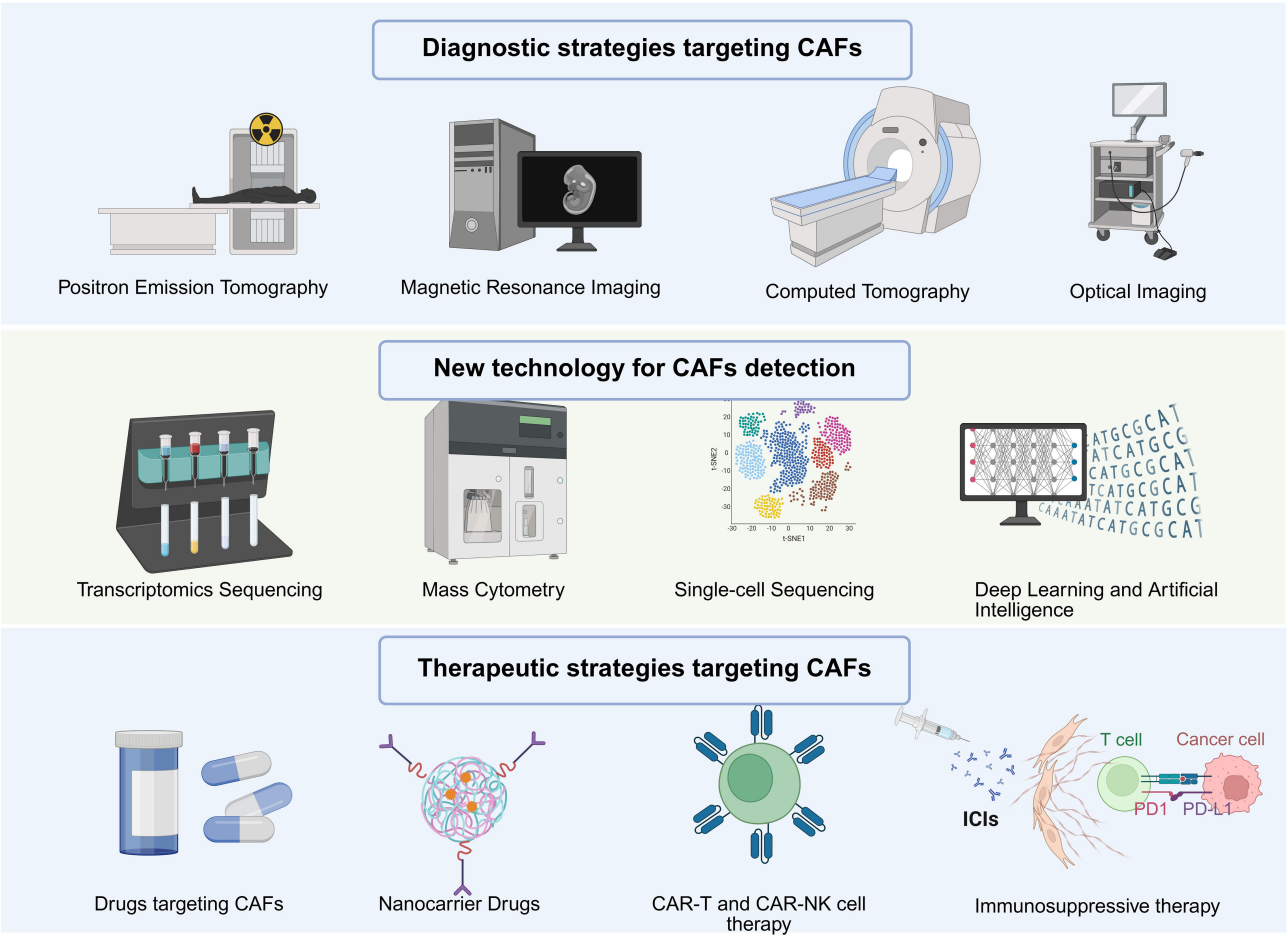
Novel diagnostic strategies for targeting CAFs. Diagnostic and therapeutic methods for CAFs, including imaging diagnosis, single-cell sequencing, deep learning, etc.

Given the high degree of heterogeneity among CAFs in different tumor types, personalized therapeutic strategies are essential. Personalized treatment strategies can also be combined with imaging technology of the tumor microenvironment, using imaging methods such as PET/MRI to dynamically monitor the distribution and activity of CAFs, and adjust the treatment plan accordingly. FAP-PET imaging technology can be used to evaluate the targeted treatment response of CAFs in real time, thereby guiding subsequent treatment decisions. In addition, integrating multi-omics data (such as single-cell sequencing, spatial transcriptomics, etc.) to deeply analyze the characteristics of CAFs in different patients will help design more accurate individualized therapies (131) (**Figure 4**).

Despite the growing interest in CAFs-targeted therapies, significant limitations remain in their clinical application. Current strategies often lack the specificity required to distinguish between tumor-promoting and tumor-suppressing CAFs subtypes, raising concerns about potential off-target effects and unintended tumor acceleration. Furthermore, CAFs populations exhibit substantial intertumoral and intratumoral heterogeneity, with tumor-specific phenotypes and functions that complicate the development of one-size-fits-all approaches. Notably, CAFs roles and abundance vary widely across cancer types—ranging from the desmoplastic stroma of pancreatic ductal adenocarcinoma to the more immune-interactive niches of breast or colorectal cancers. These variations necessitate tailored therapeutic designs that consider not only CAFs subtype identity but also their spatial organization and interaction with other stromal and immune components. Future efforts should focus on integrating spatial transcriptomics, epigenomic profiling, and high-resolution imaging to develop tumor-specific CAFs-targeting strategies that balance efficacy with safety.

## 7 Conclusion and future perspectives

CAFs are increasingly recognized as central architects of TME, mediating critical processes like ECM remodeling, immune suppression, metabolic reprogramming, and therapeutic resistance. Their pivotal role has spurred significant interest in developing CAF-targeted therapies, especially in combination with chemotherapy and immunotherapy. However, this emerging therapeutic landscape faces considerable conceptual and translational challenges requiring deeper exploration.

A primary limitation stems from the remarkable heterogeneity and plasticity of CAFs, observed both between and within tumor types. CAF subtypes exhibit diverse transcriptional profiles and functional roles—ranging from immunosuppressive (e.g., iCAFs) to mechanically supportive (e.g., myCAFs), or even immunostimulatory in certain contexts. For instance, studies in pancreatic ductal adenocarcinoma revealed that depleting α-SMA^+^ myCAFs paradoxically accelerated tumor progression and reduced survival, likely due to the loss of stromal restraints on angiogenesis and dissemination (140, 141). This exemplifies the “CAF paradox,” where not all fibroblasts are tumor-promoting; some may function as stromal gatekeepers. Furthermore, the lack of definitive and specific CAF markers severely limits therapeutic precision. Commonly used markers like FAP, PDGFRα/β, and α-SMA are not exclusive to CAFs and are expressed during tissue repair, embryogenesis, and fibrosis (142). This raises substantial risks of off-target effects and systemic toxicity, as evidenced by halted clinical trials of FAP-targeted therapies due to severe musculoskeletal toxicities in normal tissues (143).

Therapeutic development is further complicated by dynamic crosstalk between CAFs and other stromal and immune components. CAFs can reprogram dendritic cells into tolerogenic phenotypes, skew macrophage polarization toward M2 states, and physically impede T cell infiltration via ECM barriers (144). Conversely, CAFs-secreted chemokines can paradoxically enhance CD8^+^ T cell infiltration in some tumors, contradicting the prevailing view of CAFs as uniformly immunosuppressive (145). These discrepancies highlight the necessity for context-specific interpretations of CAFs function. Emerging approaches—such as CAF subtype reprogramming (instead of depletion), senolytic targeting of senescent CAFs, and combination therapies with immune checkpoint inhibitors—show promise but remain largely preclinical. Even novel strategies like CAFs-targeted CAR-T cells or bispecific antibodies face significant delivery barriers imposed by the dense fibrotic matrix and our limited understanding of the CAFs–immune–tumor axis. Imaging techniques like PET/MRI offer potential for visualizing dynamic CAFs behavior to stratify patients and track responses, but require standardization across tumor types.

In conclusion, while CAFs-targeted therapy represents a promising frontier in oncology, it must be pursued cautiously due to unresolved controversies surrounding CAFs origin, function, and context-dependent behavior. Future research should prioritize spatial multi-omics, lineage tracing, and computational modeling to decipher CAFs plasticity, validate functional biomarkers, and design safe, selective therapeutic strategies. Only by embracing this complexity can we advance from broad enthusiasm to translational precision in CAFs-directed interventions.
